# Cochlear Gene Therapy for Sensorineural Hearing Loss: Current Status and Major Remaining Hurdles for Translational Success

**DOI:** 10.3389/fnmol.2018.00221

**Published:** 2018-06-26

**Authors:** Wenjuan Zhang, Sun Myoung Kim, Wenwen Wang, Cuiyuan Cai, Yong Feng, Weijia Kong, Xi Lin

**Affiliations:** ^1^Department of Otolaryngology, Union Hospital, Tongji Medical College, Huazhong University of Science and Technology, Wuhan, China; ^2^Department of Otolaryngology, Emory University School of Medicine, Atlanta, GA, United States; ^3^Xiangya School of Medicine, Changsha, China

**Keywords:** cochlear gene therapy, review, viral-mediated gene expression, preclinical trials, hearing restoration, sensorineural hearing loss, genetic mutations, genetic deafness

## Abstract

Sensorineural hearing loss (SNHL) affects millions of people. Genetic mutations play a large and direct role in both congenital and late-onset cases of SNHL (e.g., age-dependent hearing loss, ADHL). Although hearing aids can help moderate to severe hearing loss the only effective treatment for deaf patients is the cochlear implant (CI). Gene- and cell-based therapies potentially may preserve or restore hearing with more natural sound perception, since their theoretical frequency resolution power is much higher than that of cochlear implants. These biologically-based interventions also carry the potential to re-establish hearing without the need for implanting any prosthetic device; the convenience and lower financial burden afforded by such biologically-based interventions could potentially benefit far more SNHL patients. Recently major progress has been achieved in preclinical studies of cochlear gene therapy. This review critically evaluates recent advances in the preclinical trials of gene therapies for SNHL and the major remaining challenges for the development and eventual clinical translation of this novel therapy. The cochlea bears many similarities to the eye for translational studies of gene therapies. Experience gained in ocular gene therapy trials, many of which have advanced to clinical phase III, may provide valuable guidance in improving the chance of success for cochlear gene therapy in human trials. A discussion on potential implications of translational knowledge gleaned from large numbers of advanced clinical trials of ocular gene therapy is therefore included.

## Introduction

Hearing impairment is one of the most common human disabilities. According to the World Health Organization (WHO), five percent of the world's population (~360 million people) suffers disabling hearing loss (http://www.who.int/mediacentre/factsheets/fs300/en/), defined as hearing loss >40 dB in the better hearing ear. More people are affected by severe hearing loss than the combined number of individuals affected by epilepsy, multiple sclerosis, spinal injury, stroke, Huntington's and Parkinson's diseases (Hudspeth, 1997). Hearing loss is therefore a critical public health concern, especially in aging societies. Multiple factors, including the use of ototoxic antibiotics (e.g., aminoglycosides) or cancer treatment drugs, exposures to loud noise, and genetic mutations give rise to hearing loss. Sensorineural hearing loss (SNHL), defined by the presence of deafness-causing etiologies in the cochlea and the auditory nerve, accounts for approximately 90% of all human hearing loss cases (Smith et al., 2005). Among SNHL patients, genetic factors are estimated to predispose or be directly responsible for 50–60% of all cases, with percentage higher in the developed countries (Marazita et al., 1993; Smith et al., 2005). Most cases are caused by either a single monogenic point or small indel mutation (Hilgert et al., 2009a; Hoang Dinh et al., 2009; Shearer et al., 2010). Monogenic mutations in more than 100 genes cause severe congenital or progressive hearing loss (see the Hereditary Hearing Loss website, http://hereditaryhearingloss.org, maintained by G. Van Camp ad RJH Smith). These mutations cause non-syndromic as well as syndromic hearing loss (e.g., Usher syndrome, Pendred syndrome). In addition, genetic mutations are major pre-disposition factors in age-dependent and acquired (e.g., noise- or drug-induced) hearing loss (Kokotas et al., 2007; Someya et al., 2009; Yamasoba et al., 2013; Bowl and Dawson, 2015). Genetic mutations also play a major role in common middle ear diseases (e.g., otosclerosis, http://hereditaryhearingloss.org/main.aspx?c=.HHH&n=86521).

Dramatic progress has been made in our understanding of the genetic basis of human deafness (Lenz and Avraham, 2011). Genetic diagnosis of deafness provides essential information for cochlear gene therapies, and rapid progress has been made in both the accuracy and accessibility to genetic testing in the last few years (Shearer et al., 2011; Lin et al., 2012). Early detection provides advantages for disease management and treatment. Identification of mutations in syndromic deafness genes could be many years before the emergence of symptoms in patients, giving time for planning disease management. In many instances, distinct mutations in the same gene can lead to different disease manifestations. The detailed information about pathological variants of deafness genes are found in many expertly-curated databases, such as ClinGen and ClinVar (https://www.clinicalgenome.org/), HGMD (http://www.hgmd.cf.ac.uk/ac/index.php) and many generated by academic research groups (e.g., Hereditary Hearing Loss Homepage, http://hereditaryhearingloss.org). NIH has on-going programs to support expert groups for the curation of clinically-relevant genetic variants according to the standard of the American College of Medical Genetics and Genomics (ACMG) (Richards et al., 2015; Patel et al., 2017). With continued improvements, many of these centralized databases will become more accurate and convenient sources for interpreting the meaning of genetic variants with clinical significance for human deafness. Many clinical laboratories currently already perform genetic diagnosis for multiple deafness genes (https://www.ncbi.nlm.nih.gov/gtr/all/?term=deafness).

For patients with hearing loss beyond what can be helped with hearing aids the only effective treatment option is the cochlear implant (CI). CI is the most successful sensory prosthetic device on the market. Many reports have shown that most deaf patients can understand speech in quiet environments after receiving CIs (Wilson and Dorman, 2008; Roche and Hansen, 2015). About 300,000 patients worldwide have received cochlear implants, however this only accounts for a small fraction of all deaf patients. CIs also have major limitations and weaknesses (Muller and Barr-Gillespie, 2015; Roche and Hansen, 2015; Weiss et al., 2016) such as poor pitch perception, increased difficulties in identifying characteristics in speaker voice and tonal language especially under noisy or competing voice environments, and inability to appreciate music. CIs are prosthetic devices, which accordingly demand great care of use by patients over their lifetimes. Many research groups have been improving the CI and trying to find better alternatives. For example, optogenetics based implants have been proposed to improve the sound resolution power (Moser, 2015). However, the concept of optogenetics ultimately still relies on prosthetic devices that emit controlled light stimuli inside the cochlea. A non-viral approach, using CI electrodes to transduce mesenchymal cells lining the cochlear perilymphatic canals by electroporation with DNA vectors to drive the expression of brain-derived neurotrophic factor (BDNF), is found to stimulate regeneration of neurites of SGNs toward the CI electrodes. This significantly improves neural and CI interface by lowering stimulus thresholds and expanding dynamic range of the cochlear nerve (Pinyon et al., 2014). A primary motivation in developing biological treatments is to restore hearing without the implantation of any prosthetic device, and to achieve sound resolution quality and unit cost that is much better than what is currently achievable with CIs, which have an inherent limitation of frequency resolution as imposed by inter-channel electrical interference. This review will first discuss the cellular basis of cochlear gene therapy for a variety of deafness mutations and the likely boundary conditions set by our understanding of how virally-mediated gene therapies work in the cochlea. We will then focus on the latest developments in preclinical gene therapy studies in animal models, and summarize the major remaining obstacles that need to be resolved before advanced human trials can begin. We will also discuss likely strategies that may facilitate more advanced stages of clinical trials of cochlear gene therapy for SNHL. Cell therapies of SNHL are not the topic of this review. Interested readers should find many other excellent reviews (Brigande and Heller, 2009; Muller and Barr-Gillespie, 2015).

## Design of gene therapy strategies depends on cellular mechanisms affected by mutations

**Figure 1:**
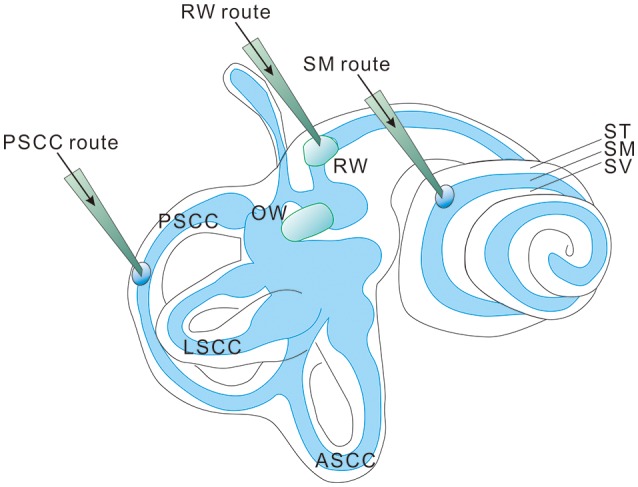
Three injection routes commonly used in cochlear gene delivery. PSCC, posterior semicircular canal; LSCC, lateral semicircular canal; ASCC, anterior semicircular canal; RW, round window; OW, oval window; ST, Scala tympani; SM, Scala media; SV, Scala vestibuli.

Most hereditary hearing loss is caused by homozygous recessive mutations (Lenz and Avraham, 2011; Shearer et al., 2013) and the deafness genotype-phenotype relations usually are tightly defined (Smith et al., 2005). This means that most cases of genetic hearing loss are potentially amenable to gene replacement or augmentation therapy by exogenous expression of a single wildtype (WT) gene (Sacheli et al., 2012) using three commonly used injection routes into the inner ear (Figure 1; Sacheli et al., 2012; Suzuki et al., 2017; Yoshimura et al., 2018). For dominant mutations, a recent study using a CRISPR-Cas9 genome-editing approach showed that hearing thresholds were improved in a mouse model of dominant deafness caused by a mutation in transmembrane channel-like gene family 1 gene [*Tmc1*, Beethoven (Bth) mutation, or *Tmc1*^*Bth*^ allele] (Gao et al., 2017). The targeted correction was highly precise at a resolution of just a single base pair. Injection into the neonatal cochlea of *Tmc1*^*Bth*/+^ mutant mice substantially reduced progressive hearing loss. Although there are still many translational issues which need to be overcome, these results support the applicability of cochlear gene therapies, if appropriately designed, for both recessive and dominant mutations. Cochlear gene therapy may also provide neurotrophic or other protective (e.g., anti-apoptosis) functions for the survival of cochlear sensory and non-sensory cells (Fukui and Raphael, 2013). This approach may not be gene or mutation specific, but rather based on our understanding of the interplays of important biological pathways for normal cochlear functions.

In addition to the inheritance patterns of genetic mutations, effective cochlear gene therapy also critically depends on our understanding of the specific molecular/cellular mechanisms of defects caused by different genes and by various types of mutations within the same gene. Monogenic mutations affecting the functions of hair cells, supporting cells, or the stria vascularis (SV) are three major types of mutations causing severe hearing loss (Hilgert et al., 2009a; Avraham and Kanaan, 2012). Deafness genes encode proteins with a wide range of molecular functions vital for cochlear functioning, such as development of the sensory organ, sound transduction in the stereocilia of hair cells, maintenance of the endocochlear potential (EP) and high concentration of extracellular potassium, and synaptic neurotransmission between hair cells and spiral ganglion neurons (SGNs). Major proteins made from deafness genes include ion channels and transporters, gap junctions and tight junctions, protein subunits in cytoskeleton and molecular motors, and transcription factors transiently expressed in cochlear development (Steel and Kros, 2001; Dror and Avraham, 2009). Whether a mutation affects early cochlear development and leads to significant cellular degeneration is a major factor in determining the “treatment time window.” Hearing loss has also been linked to mutations in genes in the mitochondrial genome and in nuclear genes regulating mitochondrial functions (Guan et al., 1998; Kokotas et al., 2007). Acellular structures in the cochlea such as tectorial membranes (with collagens and tectorins as major components) could also be affected by gene mutations (Verhoeven et al., 1998). The error tolerance of the auditory transduction organ, the cochlea, appears to be one of the lowest among all the organs in the body. This has been suspected to account for the fact that most genetic deafness cases are non-syndromic (Dror and Avraham, 2009). Diseases associated with loss-of-function mutations can generally be treated by gene replacement/supplementation therapy, whereas those associated with gain-of-function mutations require eradication of mutant alleles in addition to supplementation of the normal copies of the gene (Gao et al., 2017).

Pathogenic process, pattern and time course of degeneration in the cochlea for specific deafness genes may suggest which mutation(s) could be reasonably expected to have a higher chance of success in cochlear gene therapy human trials, which merits more detailed discussion here based on preclinical studies obtained from animal models (Table 1).

**Table 1:** A summary of representative pre-clinical studies of cochlear gene therapy using mutant mouse models.

**Animal model**	**Viral vector**	**Injection method and time**	**Ave. ABR improvement (@best freq.) & treatment efficacy duration**	**Targeted cells & major morphological improvement**	**References**
*Vglut3^−/−^* mice	AAV1-VGLUT3	Time: P1-3 & P10. Route: RWM injection & Cochleostomy Delivery.	~30 dB by tone burst, ~60 by click ABR. Lasted for 3–6 months depending on injection time.	IHC. Morphological improvement observed at the ultracellular level.	Akil et al., 2012
*Kcnq1^−/−^* mice	AAV1-kcnq1	Time: P0-P2 Route: injection into the scala media	~60 dB (control (ctrl) used was 90 dB), lasted for 4–6 months	SV marginal cells. Correction of the collapse of the Reissner's membrane and degeneration of HCs and cells in the spiral ganglia	Chang et al., 2015b
*MsrB3^−/−^* mice	rAAV2/1-MsrB3-GFP	Time: E12.5 Route: Injection into the otocyst using the transuterine approach	~70 dB (ctrl used was 100 dB).	IHCs and OHCs. Restoration of stereociliary bundles	Kim et al., 2016
Lhfpl5/Tmhs^−^	exo-AAV1-HA-Lhfpl5	Time: P1-P2 Route: RWM injection and by cochleostomy at the basal turn.	~30 dB (ctrl used was 100 dB).	Improved IHC and OHC survival. *In vitro* FM1-43 loading assay showed increased HC function.	Gyorgy et al., 2017
Usher1c (c.216G>A)	AAV2/Anc80L65. CMV.harmonin and others	Time: P0–P1 and P10–P12. Route: RWM	~50–60 dB (ctrl used was 110 dB) for mice injected at P0-P1. Efficacy lasted for 6 months, which is longest time point tested.	Improved IHC and OHC survival. *In vitro* FM1-43 loading assay showed increased HC function.	Pan et al., 2017
TMC^−/−^	AAV2/1-Cba-Tmc	Time: P0–P2. Route: RWM	~20-30 dB (ctrl used was 110 dB).	Transduction current at the single-cell level was preserved in hair cells of injected *Tmc*-deficient mice.	Askew et al., 2015
conditional *Gjb2* knockout mice	AAV-CB7-Gjb2-GFP	Time: P0-P1 Route: scala media injection	0 dB	Cx26 expression was restored and ectopically expressed in several cell types. Cochlear gap junctions (GJs) were re-established. Both cell death in the organ of Corti and degeneration of SGNs were substantially reduced.	Yu et al., 2014
Gjb2 conditional KO mice Cx26fl/flP0-Cre	AAV5-Cx26	Time: P0 and P42 Route: RWM	0 dB when injection was made at P42. ~30 dB when treated on P0, unclear how long the treatment effects lasted.	No morphological improvement when treated on P42. Proper formation of the tunnel of Corti and preservation of IHCs and OHCs, as well as SCs and SGs were observed when treated at P0.	Iizuka et al., 2015
Whrn^wi/wi^ mice	AAV2/8-whirlin	Time: P1~P5 Route: injection into the posterior semicircular canal.	~20 dB at 8 kHz. Significant vestibular function preservation observed. Treatment effects last for about 4 months.	IHC expression of whirlin and its transportation to stereocilia tips were restored. The length of stereocilia was fully or partially restored. The stereocilia architecture was also improved. IHCs survival was increased, but only temporarily.	Isgrig et al., 2017

*Those in shaded rows are studies in which only morphological and no significant hearing improvements are observed. More complete and detailed information obtained from genetic mouse models, as well as pharmacologically-induced and noise-induced mouse models, is given in Supplemental Table 1. RWM, round window membrane*.

### Mutations mainly affect non-sensory cells in the cochlea

**Figure 2:**
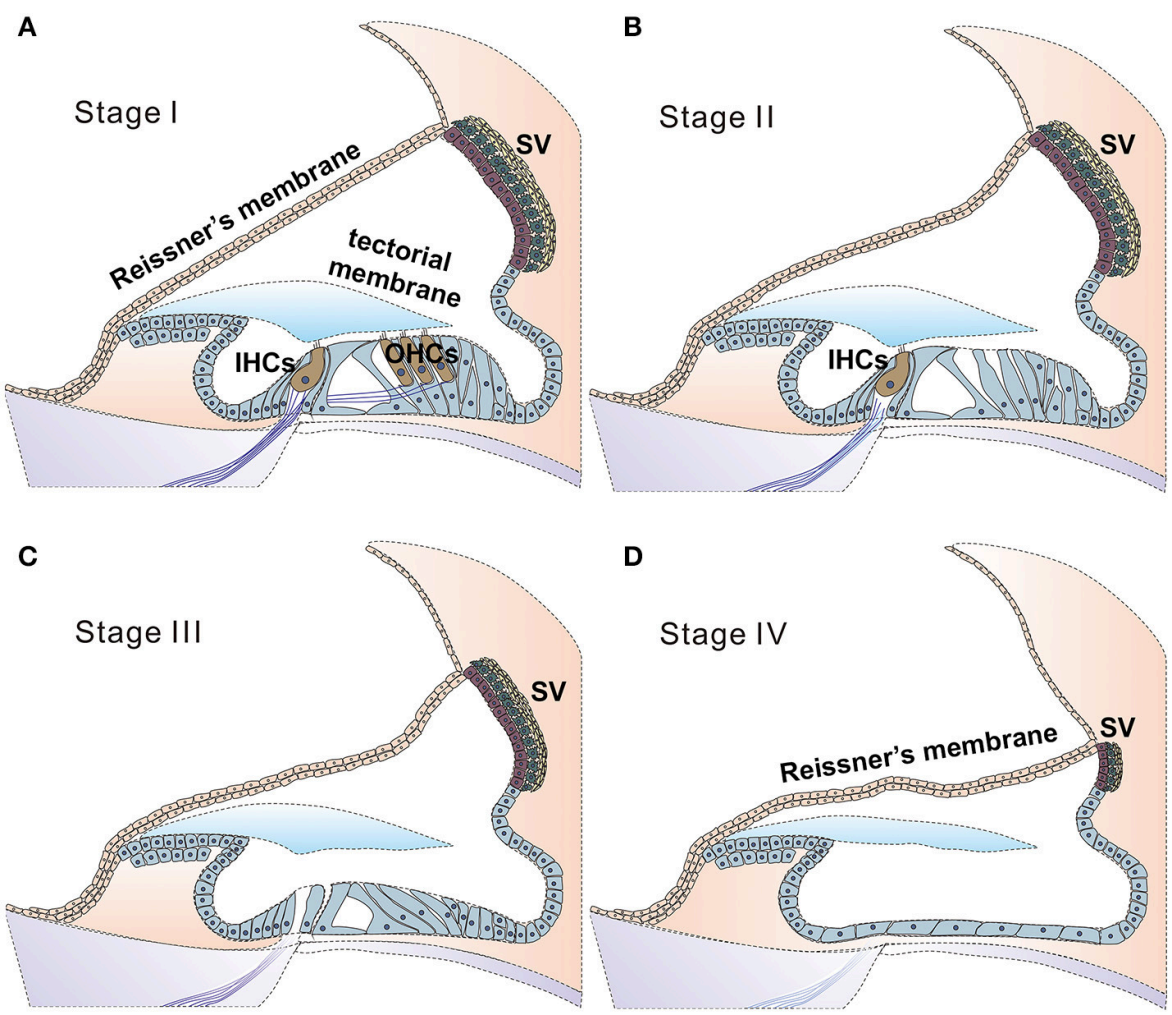
Stages of degeneration in the cochlea may greatly affect the likelihood of success for cochlear gene therapies. Illustration of proposed four stages of disease progression in the cochlea caused by genetic mutations, as suggested by ocular gene therapy studies (Dalkara et al., 2016). Healthy cochlea is composed of health sensory cells (both IHCs and OHCs), supporting cells and cells in the SV. Deafness mutations may give rise to no significant cell death (**A**, defined as stage I), cell death primarily in (or started from) outer HCs (**B**, stage II), followed progressively and more severe degeneration stages of III (**C**, both inner and outer HCs are lost) and IV (**D**, all sensory and supporting cells are lost, leaving a layer of non-specific epithelial cells in the organ of Corti. SV is severely degenerated). According to results obtained from human clinical trials of ocular gene therapy, diseases at the stage III or stage IV have little chance for a successful outcome for gene therapy (Dalkara et al., 2016). In these cases, new strategies for applying gene therapy to restore hearing will need to be explored, and one is suggested in Figure 3.

Findings in connexin30 (Cx30, *Gjb6*) knockout mice (Teubner et al., 2003) indicate that absence of an endocochlear potential (EP) during cochlear development is the major cause of hearing loss. Time courses of cellular degeneration in the cochlea of *Gjb6* null mutant mice are much slower than that of cochlea in *Gjb2* (gene name for connexin26, Cx26) null mice (Sun et al., 2009). Our observations made from conditional connexin26 (cCx26) null mice (Wang et al., 2009; Chang et al., 2015a) show that *Gjb2* null mutations predominantly affect the normal development of the sensory epithelium in the cochlea before the onset of hearing. In contrast, normal cochlear functions at the adult stage do not require normal *Gjb2* expression in mice (Chang et al., 2015a). The data obtained from our lab and others (Chen et al., 2014) do not support the K^+^ recycling hypothesis, which speculate that *GJB2* mutations cause deafness by disrupting K^+^ recycling in the cochlea. Based on these results, we have proposed (Chang et al., 2015a) that the absence of Cx26 in supporting cells of the organ of Corti during the critical postnatal period in mice may greatly reduce the intercellular diffusion of molecules essential for normal cellular activities (e.g., glucose; Chang et al., 2008), and may hinder the postnatal maturation of the organ of Corti in cCx26 null mice. The non-functional sensory epithelium at multiple cellular levels may lead to massive degeneration mainly in the middle and basal turns of the cochlea, as we have observed in mice (Wang et al., 2009; Chang et al., 2015a). For pendrin (gene name: SLC26A4) null mutations, studies in mice again (Choi et al., 2011; Li et al., 2013) indicate that pendrin expression in the cochlea is required only during a narrow time window in the early development period (E16.5 to P2), but not for the maintenance of normal hearing later (Choi et al., 2011). Treatments performed later than this time window may have to deal with severe degeneration in the organ Corti first, which may progress beyond the stage III (Figure 2C). This may significantly diminish the chances of success for cochlear gene therapy of Pendrin mutations.

**Figure 3:**
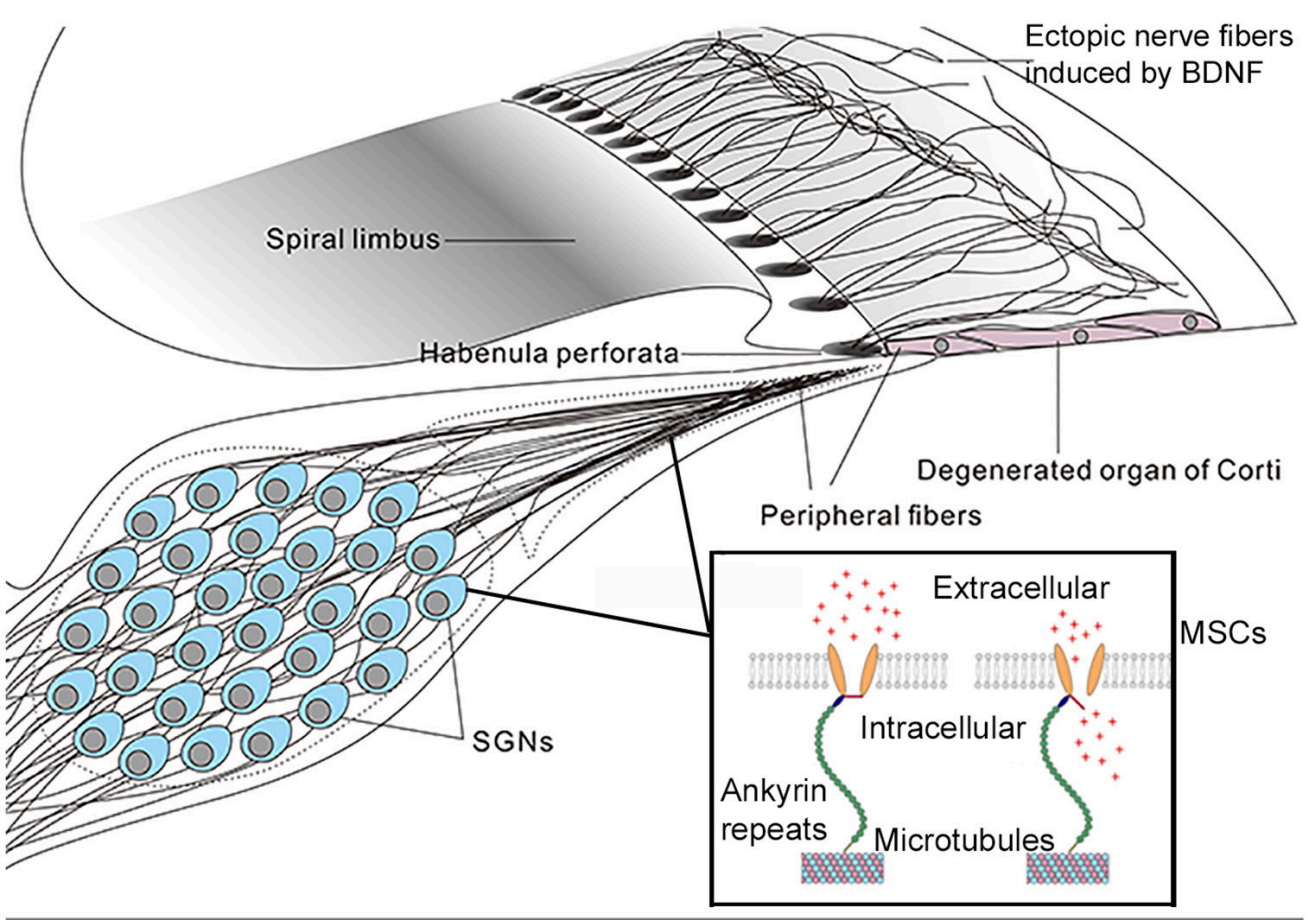
A mechano-genetics approach for treating SNHL. Degeneration in the cochlea caused by many deafness gene at the adult stage will be in stages III and IV (Figure 2), which is treatable by CIs that directly excite SGNs by extracellular electrical field potential. This figure gives an illustration of possible outcome in degenerated organ of Corti after receiving gene therapy to express both neurotrophic (e.g., BDNF) and mechano-sensitive channels (MSCs). Peripheral fibers of survived SGNs are supposed to be induced to grow into the area of remaining basilar membrane. The MSCs are virally-expressed in the cell membrane of SGNs. These MSCs are normally attached to microtubes via ankyrin repeats (boxed insert), and the MSCs are opened directly by mechanical stimuli to the cell membrane (Zhang et al., 2015). It is hypothesized, as an alternative gene therapy method, that virally-expressed MSCs may render SGNs directly respond to vibration of the basilar membrane by firing action potentials. The advantage of this approach is that it requires similar cellular survival condition as that in the cochlea of CI patients.

Most deafness genes individually affect relatively few patients and are often concentrating in a few related families and specific geographic locations (Tekin and Arici, 2007; Yuan et al., 2009). In contrast, mutations in a small number of deafness genes (e.g., *GJB2, GJB6, SLC26A4, TMC1 etc.)* are responsible for causing a large percentage of genetic deafness cases. By some estimates >60% of genetic deafness cases are caused by mutations in *GJB2* and *SLC26A4* genes alone (Denoyelle et al., 1999; Yuan et al., 2009; Brownstein et al., 2011). Successful gene therapy applied to these few genes may potentially benefit a larger proportion of deaf patients. However, it seems that these genes all affect the development of the cochlea. If the results obtained from mouse models (Inoshita et al., 2008; Wang et al., 2009; Chen et al., 2014) could be applied to humans, these imply that cochlear gene therapy for *GJB2, GJB6*, and *SLC26A4* will need to be performed early enough to restore normal hearing, perhaps in embryonic stage in humans. For a non-lethal disease, whether treatment with inherently high risks can be or worth to be performed to human fetus is debatable (David and Waddington, 2012). In these cases, new strategies for applying gene therapy to restore hearing will need to be explored (Figure 3).

### Mutations mainly affect various functions of hair cells (HCs)

A large variety of deafness genes (Hilgert et al., 2009a) play essential roles in sound transduction (e.g., *TMC1)*, the development or maintenance of the hair bundles [e.g., myosin VI (*MYO6*), myosin VIIA *(MYO7A)*, myosin XVA *(MYO15A)*, cadherin 23 *(CDH23)*, and Protocadherin-15 *(PCDH15*)], or for neurotransmission [e.g., Otoferlin (*OTOF)*, pejvakin (*PJVK*)] at the base of HCs. Antisense oligonucleotides were used in a pioneering study by Lentz et al. (2013) to correct defective pre-mRNA splicing of transcripts resulting from the a c.216G>A mutation in the *USH1C* gene. The novel treatment designed with the use of a relatively small molecule results in normal protein expression, improved stereocilia organization and survived cochlear hair cells. Functional studies reveal significant preservation of both the vestibular and hearing functions in mice. Using an Ush1c c.216G>A knock-in mouse model to study the Usher typeI C disease, Pan et al. (2017) tested whether cochlear gene therapy could be used to target hair cells to correct the deafness phenotype. A novel synthetic Adeno-associated virus (AAV), Anc80L65 which is the first AAV serotype to transduce outer HCs efficiently, was used and this viral vector was able to transduce >90% of HCs. Anc80L65 was found in another independent study to show excellent transduction efficiency, even for adult hair cells (Suzuki et al., 2017). The treatment (Pan et al., 2017) demonstrates morphological preservation in the cochlea, and the auditory thresholds were improved for 60–70 dB compared to untreated ears when recombinant viral vectors were injected at P0-P1 through round window membrane into the scala tympani. The same injections performed at P10-P12 didn't yield any treatment effects, again suggesting that a window of opportunity for treatment only exists at early postnatal stage in mice. The positive treatment effect appears to last at least 6 months, as determined by hair cell survival in the cochlea. Its immunogenic profile in human will need to be characterized before clinical trials could start. In another study using Sans null mutant (*Ush1g*^−/−^) mice (Emptoz et al., 2017), AAV-mediated expression of *Ush1g*, which is a submembrane scaffold protein critical for the morphogenesis of the stereocilia bundle, was found to preserve hair bundle functions to maintain both hearing and balance functions to near wild-type level. Results of these studies indicate that virally-expressed proteins can be expressed in hair cells. When recombinant viral vectors are injected before maturation of hair cells, even severe dysmorphogenesis of stereocilia bundles can be prevented, suggesting that virally-expressed proteins were transported to the correct location of hair bundle to exert therapeutic effect. These studies suggest cochlear gene therapy could be a powerful tool to treat genetic mutations that specifically affect hair bundle functions, if the therapy is delivered before hair cells are degenerated. The best treatment time window for mutations in this category seems to be before HCs degenerate. Long-term viral expression of a WT gene in HCs may replace or enhance the defective gene, especially for those transiently needed in development, and a stable therapeutic effect may potentially be obtained. If hair cells are already degenerated but sufficient numbers of supporting cells are still present, then a master transcription factor (such as protein atonal homolog 1 (*ATOH1*)) may be expressed in the supporting cells to induce trans-differentiation of supporting cells into the hair cells. However, the effects of genetic mutation will also need to be corrected in trans-differentiated supporting cells. This may require the co-expression of more than one gene in targeted cell populations.

### Mutations mainly affect development or function of the SV

The establishment and maintenance of the EP depend on a chain of ion channels and transporters working together in the cell membranes of basal cells, intermediate cells (e.g., ATP-sensitive inward rectifier potassium channel 10 (*KCNQ10*)) and marginal cells (e.g., K_v_7.1 potassium channel protein (*KCNQ1*)), for a review see (Lang et al., 2007). Mutations in any one of these key membrane proteins in the SV results in loss of the EP and causes deafness. For example, mutations in the *KCNQ1* (also known as *KvLQT1* or *Kv7.1*) or *KCNE1* (coding for E regulatory subunit 1 of a potassium voltage-gated channel subfamily) cause Jervell Lange-Nilsen (JNL) syndrome (Jervell and Lange-Nielsen, 1957). Requirements for performing gene therapy to correct mutations in SV cells are distinctively different from those required for treating sensory hair cells. The SV is a hard-to-access space for gene delivery, especially at adult stage when hearing damage resulting from surgical procedure needs to be avoided. Effective inoculation usually requires injection of viral particles directly into the scala media (Fukui and Raphael, 2013; Wang et al., 2013). Intermediate and basal cells are especially hard to reach for viral vector mediated expressions. No successful reports have been published so far. This will be a barrier to overcome if deafness genes in these cells (e.g., *KCNJ10*) need to be considered for cochlear gene therapy. No reports have indicated that injections into the scala tympani, or posterior vestibular semi-circular canal, have resulted in efficient viral expression in any of the three types of cells in the SV. Inoculation into the posterior semicircular canal at the adult stage, while successful in transducing HCs (Suzuki et al., 2017), is ineffective for transducing any SV cells. In addition, cells in the SV (e.g., intermediate cells) may slowly turnover (Conlee et al., 1994). Cell renewal may be disadvantageous for stable and long-term AAV expressions. Different AAV serotypes may have vastly different efficacy in the transduction of various types of cochlear cells. More detailed information can be found in these reviews (Sacheli et al., 2012; Ahmed et al., 2017).

One successful cochlear gene therapy report for correcting *Kcnq1* null mutation in the marginal cells has been reported (Chang et al., 2015b). AAV1 expressing *Kcnq1* was injected postnatally (P0–P2) into the endolymph, which resulted in *Kcnq1* expression in about 70% cochlear marginal cells where the native *Kcnq1* is exclusively expressed. Examination of cochlear morphology shows that the collapse of the Reissner's membrane, degeneration of HCs and SGNs are prevented. Functional studies show normal EP in treated ears and nearly-normal auditory brainstem responses (ABRs). Significant hearing improvements last for about 6 months in treated *Kcnq1*^−/−^ mice (Chang et al., 2015b). In the future, this approach may be used to test the feasibility of treating other inherited deafness cases in which the SV is the predominant site affected (e.g., mutations in *KCNE1*, coiled-coil domain containing 50 protein (gene name *CCDC50*), grainyhead like transcription factor 2 (gene name *GRHL2*), transmembrane serine protease 3 (gene name *TMPRSS3*).

### Mutations mainly affect neurotransmission between HCs and SG neurons that result in the auditory neuropathy

Auditory neuropathy (AN) is a type of hearing impairment in which neural transmission between the hair cells and SGNs at either the pre- or post-synaptic sites are impaired (Yasunaga et al., 1999; Delmaghani et al., 2006; Seal et al., 2008). By some estimates this type of mutations may account for about 10% of all cases of congenital hearing impairment (Starr et al., 2000). Discussions given above clearly suggest the theoretical basis for treating pre-synaptic types of auditory neuropathy, since virally-mediated gene expressions in almost 100% HCs are possible (Seal et al., 2008; Pan et al., 2017). In addition, studies have demonstrated that virally expressing genes in SG neurons is feasible (Sacheli et al., 2012), supporting the possibility of treating post-synaptic types of AN, as long as the gene product (e.g., *pejvakin*) expressed from recombinant viral vectors can be efficiently transported to the peripheral terminals of the SG neurons.

One diagnostic hallmark of the AN is the presence of relatively intact outer hair cells (OHCs) as determined by the normal distortion product otoacoustic emission (DPOAE) measurements. Studies in mouse models show that mutations result in the AN spectrum of diseases usually do not lead to severe cellular degeneration in the cochlea (Delmaghani et al., 2006; Akil et al., 2012). Many stage I gene candidates in the Table 2 belong to this category. From the point of view of morphological preservation in the cochlea, AN mutations could be the best candidates to conduct adult-stage cochlear gene therapy trials since large number of HCs and SGNs are still present, although peripheral branch of the SGNs may be withdrawn from their targets. On the other hand, AN mutations (e.g., *pejvakin;* Delmaghani et al., 2006) may also affect neurons in the upper central auditory centers, which makes localized cochlear gene therapy unlikely to be successful if the defects in the central auditory system are left untreated. Another significant hurdle in targeting some of the AN genes (e.g., *OTOF*, ~6.1 kb) is the gene size. The maximum packaging limit of AAVs, which is the most widely-used vector for clinical trials so far (Dalkara et al., 2016), is usually below 5 kb. It has been suggested that a dual vector approach, which has been used to delivery genes >5 k bp in ocular gene therapy studies (Ghosh et al., 2011; Dyka et al., 2014), could be used to overcome this limitation. Adenovirus and lentivirus don't have the packing limit of 5 kb. However, they are much less common in human clinical trials, due to long-term safety concerns (Sacheli et al., 2012).

**Table 2:** Degeneration stages as defined in Figure 1 in the adult cochlea of mutant mouse models.

**Degeneration stage in adult**	**Gene name**	**Main cellular expression sites in the cochlea**	**Major morphological findings in the cochlea of mutant mice**
Stage I^*^	*STRC*	An extracellular matrix protein that attaches the tallest stereocilia of the OHC to the tectorial membrane	Tip links are still present, however horizontal top connectors are absent from the hair bundles of the OHCs. The distal ends of the stereocilia are more loosely connected than in wild-type mice (Verpy et al., 2011).
Stage I	*CLDN11*	Tight junction protein of SCs and HCs (Gow et al., 2004; Hilgert et al., 2009a)	In mice, mutations in *Cldn11* do not change the EP. Both OHC and IHCs are intact for the first few months, then degeneration starts slowly in OHCS, followed by IHC degeneration (Gow et al., 2004; Hilgert et al., 2009a).
Stage I	*TECTA*	non-collagenous component of the tectorial membrane (TM)	TM is detached completely from the organ of Corti (Legan et al., 2014).
Stage I or II	*OTOF*	At the synaptic cleft of the IHC (Egilmez and Kalcioglu, 2016)	Defective synaptic vesicle fusion at the IHC ribbon synapse. OHCs are less affected. In *Otof* null mice, OHC function is preserved while IHC synaptic exocytosis is abolished (Hilgert et al., 2009b). Central auditory neurons are also affected (Yasunaga et al., 1999).
Stage I or II	*PJVK*	In HCs and SGNs	OHC degeneration and followed by delayed loss of IHCs (Delmaghani et al., 2006).
Stage II	*KCNQ4*	IHCs, OHCs and SGNs	Mainly manifest as OHC degeneration and loss of function (Boettger et al., 2002).
Possibly at stage II	*GJB6*	SCs, but not expressed by HCs.	Co-assembled with the *Gjb2*. Comparing to the *Gjb2* null, *Gjb6* null show delayed time course of degeneration starting from OHCS (Sun et al., 2009). Severe degeneration of SCs & HCS in the middle & basal turns eventually is observed after a few months in mice.
Stage III or IV	*GJB2*	SCs, not expressed by HCs	The cochlea is not fully developed. Severe degeneration of all types of cochlear cells in the middle & basal turns at onset of hearing in mice. HCs and SCs survive in the apical turn, but with immature functional features (Wang et al., 2009).
Stage III	*TMC1*	In both IHCs, OHCs and neurosensory epithelia of the vestibular organs.	Hair cell degeneration, and secondary degeneration of other cochlear cells, staring from the onset of hearing in mice (Kawashima et al., 2011; Pan et al., 2013).
Stage III	*CDH23*	A main component of the tip link	Hair cell degeneration, and secondary degeneration of other cochlear cells early in cochlear development in mice (Di Palma et al., 2001).
Stage III	*USH1C*	In stereocilia, the cuticular plate, the lateral plasma membrane and synapses.	Hair cell degeneration, and secondary degeneration of other cochlear cells early in cochlear development in mice (Hilgert et al., 2009a; Lentz et al., 2013).
Stage III	*MYO3A*	at stereocilia tips, also found further down the shaft of the stereocilia	Hair cell degeneration, and secondary degeneration of other cochlear cells (Hilgert et al., 2009a).
Stage III	*MYO6*	in the cuticular plate	Hair cell degeneration, and secondary degeneration of other cochlear cells early in cochlear development in mice (Friedman et al., 1999).
Stage III	*MYO7A*	Mainly in the stereocilia but also along the lateral membrane of the HCs, in the cuticular plate and in the synaptic region.	Malformation of hair cell stereocilia and progressive degeneration of HCs in the organ of Corti early in development stages (Zuo, 2002).
Stage III or IV	*SLC26A4*	In the apical membrane of outer sulcus and spiral prominence epithelial cells that border the endolymph, in the SG and in SCs	Degeneration of all types of cells in the cochlea, starting from embryonic stage (Wangemann et al., 2007).
Stage III or IV	*KCNQ1*	Apical membrane of the marginal cells of the stria vascularis.	Collapse of Reissner's membrane, degeneration of IHC, OHCs and other cells in the organ of Corti, degeneration of cells in the SV (Casimiro et al., 2001; Chang et al., 2015b).
Stage III (possibly II)	*ATP6V1B1*	In the epithelial cells of the endolymphatic sac and duct, and in the interdental cell layer of the cochlear spiral limbus	Enlargement of endolymphatic sac and duct. Some HCs and SGNs are preserved. OHCs were generally absent in the cochlear base, but present to a variable extent in the apex (Karet et al., 1999).

*Representative examples are given here due to space and formatting limitations. More complete and detailed information of deafness genes is provided in Supplemental Table 2*.

* *Definition of degeneration stages are given in the Figure 2. Our discussions suggest that the chance of success of cochlear gene therapy is greatly affected by the severity of cochlear degeneration. Only disease progression with less severe stages (stages I&II, given in shaded rows) appears to be amenable to cochlear gene therapies for hearing restoration*.

## The promises and challenges of pre-clinical cochlear gene therapy trials

Mouse models are the most popular in pre-clinical trials of cochlear gene therapy for many reasons. There are striking similarities between humans and mice in proteins essential for hearing (Muller and Barr-Gillespie, 2015). The two species share many deafness genes and pathogenic mutations and they have been confirmed by data from both human families and mutant mouse models. The cochlea is highly compartmentalized and separated from the rest of the body by the blood-cochlear barrier (BCB), which minimizes the therapeutic injection volume and leakage into the body's general circulation system, to protect cochlear immune privilege and reduce the chance of systemic adverse immune responses. As the hair cells and supporting cells in the cochlea normally do not divide, the cells in the cochlea remain stable, therefore making it possible to use non-integrating viral vectors (e.g., AAV) for sustained transgene expression (Sacheli et al., 2012; Dalkara et al., 2016). AAV appears to be a promising virus for cochlear gene therapies based on results obtained in human trials of ocular gene therapy (Dalkara et al., 2016). The reasons for the success of AAV in human ocular gene therapy include: (1) proven safety profile-a large number of human trials have shown that AAV lacks pathogenicity and possess very low immunogenicity. (2) long-lasting transgene expression in non-dividing cells (Colella et al., 2009). (3) the small size of AAV (~20 nm, which is five times smaller than Adenoviruses) helps the diffusion across cellular barriers to reach targeted cells. Many AAV subtypes can provide gene delivery to hair cells without the difficult route of scala media injections (Akil et al., 2012; Yoshimura et al., 2018). On the other hand, the disadvantage of AAVs is that they have a packaging limit of about 5 kb, which limits its application when larger genes (e.g., many of the Usher genes) need to be delivered. This limitation, however, may be overcome by using a two-vector approach (Xu et al., 2004; Ghosh et al., 2011).

Preclinical studies are similarly characterized by the presence of both advantages and unique challenges; the former are conferred by the technical benefits offered by mouse models, and the later center on difficulties inherent to conducting trials in the peripheral auditory transduction organ. The contralateral ear can be used as the same-animal control, which is the best control possible and especially helpful in evaluations of long-term treatment outcomes. As a cautionary note, there are reports of leakage into the other side of the ear when injections are made into the scala tympani (Lalwani et al., 2002). Common non-invasive and objective tools for functional tests are applicable to both animal models and humans, such as the ABR and DPOAE tests, computed tomography (CT) and magnetic resonance imaging (MRI) of the cochlea. These shared methodologies offer great value in both diagnostics and follow-up examinations. On the other hand, the unique anatomical features and the extreme sensitivity of the mechano-transduction organ of the cochlea pose some formidable challenges in cochlear gene therapy studies. Many surgical operations are likely to induce significant hearing loss, especially at the adult stage (Wang et al., 2013). These may include injections through the round window, stapes, and cochlear bony walls into the scala tympani or scala media (Sacheli et al., 2012). The difficulty of delivery into the cochlea without causing significant hearing loss at the adult stage is one of the central issue to solve for advancing cochlear gene therapy into human trials. Recent publications have suggested promising approaches (Suzuki et al., 2017; Tao et al., 2018; Yoshimura et al., 2018) for adult stage delivery to HCs. The semicircular approach has been suggested as a promising injection route for future cochlear gene therapy in human trials (Suzuki et al., 2017; Yoshimura et al., 2018) since the posterior semicircular canal also appears to be accessible in humans. It would be interesting to find out whether such an approach can be successfully used in adult animal models to recover hearing thresholds after severe hearing loss has occurred.

Preclinical studies of cochlear gene therapy are generally conducted to investigate: (1) Whether the virally-expressed gene is correctly transported to the appropriate intracellular location and assembled with the right molecular partner(s), or whether the natively expressed partner works together with the virally expressed protein to perform the needed cellular functions in the long-term. (2) As virally-expressed protein is often driven under the control of strong generic promoter, it is important to investigate whether there is any significant negative effect from over-expression of the gene, and from ectopically-expressed proteins in the cochlea, in both the short- and long-term. (3) For many deafness genes transiently expressed during cochlear development, it is important to study whether virally-expressed proteins have expression dynamics appropriate for normal cochlear development and whether the absence of down-regulation from the viral expression poses a problem for the normal cochlear functions in the mature stage. (4) Whether the concept of gene replacement, enhancement or correction works effectively in animal models for a specific deafness gene and for a specific type of mutation, and whether the treatment effects are long-lasting. If yes, what are the reasons, and how then to achieve longer term treatment effects? (5) Are significant adverse immuno-reactivities and other safety issues absent when tested in different species? Absence of adverse effects in mice does not guarantee the same for larger animals, therefore tests done with different animal models (e.g., non-human primates) may be needed for this purpose. Following section shows that significant progresses have been made in answering many of these questions.

## Lessons learned from clinical ocular and pre-clinical cochlear gene therapy studies

Sixteen years after the landmark study by Acland et al. showing successful gene therapy for dogs with Leber's congenital amaurosis (LCA) (Acland et al., 2001), the field of ocular gene therapy is making rapid advances (Garoon and Stout, 2016; Sengillo et al., 2016). Currently, most ocular gene therapy trials are directed toward inherited retinal dystrophies of photoreceptors and retinal pigment epithelium (e.g., LCA, choroideremia, Stargardt disease), as well as for age-related exudative macular degeneration (AMD) (Dalkara et al., 2016; Garoon and Stout, 2016; Campa et al., 2017). A search on clinicatrials.gov for these diseases found that virtually all current (as of April of 2018) human gene therapy trials use AAV for therapeutic delivery, suggesting that AAV-mediated expression is the major treatment option and non-viral delivery is still far from clinical applications of ocular diseases. Many studies have advanced to clinical phase I-III trials (Dalkara et al., 2016; Garoon and Stout, 2016; Campa et al., 2017). A recent internet search (conducted in Jan 2018 on clinicaltrials.gov) using the term “ocular gene therapy” found 93 total clinical trials ongoing worldwide, and 6 have already advanced to phase III. Clinical trials for LCA using AAV2 for both adult (>18 years) and young patients (7–18 years) have shown results with significant improvement in full-field light sensitivity threshold tests (Jacobson et al., 2012). In December of 2017, Philadelphia-based Spark Therapeutics Inc. obtained the first FDA approval of LUXTURNA™ for gene therapy to treat retinal dystrophy due to a mutation in the retinoid isomerohydrolase made from the *RPE65* gene (Dias et al., 2018).

Gene therapy for ophthalmologic diseases is far more advanced than for SNHL. This may be partially explained by earlier success in pre-clinical trials of large animal models (Acland et al., 2001). The field of ocular gene therapy is also helped by easier access to make injection into the retina, which greatly enhance the efficiency of experimental trials. Many successful reports of pre-clinical trials in preventing genetic deafness have increased the confidence of moving cochlear gene therapy forward into human trials, with one already underway (ClinicalTrials.gov Identifier: NCT02132130). The cochlea bears many similarities to the eye, therefore experience gained in ocular gene therapy may be gleaned. One important lesson learned from ocular gene therapies is that treatment failure could be due to inefficient vector transduction or the timing of rescue in relation to disease onset (Cepko and Vandenberghe, 2013; Wert et al., 2014). Regardless of interventions occur pre- or post-onset of disease, the treatment success appears to critically depend on sufficient number of surviving photoreceptors (Davis et al., 2013). Ocular gene therapies have universally indicated that a minimal amount of surviving sensory cells is needed for a successful outcome (Figure 2). Advanced stages of retinal degeneration dramatically diminishing the chances of successful treatment. None of the clinical trials are targeted to treat retinal diseases beyond stage II as defined by Dalkara et al. (2016). Which deafness gene or specific mutations may have a realistic chance to be treated first by cochlear gene therapy to either prevent or reverse hearing loss? The accumulated knowledge base for the development of ocular gene therapy may provide some hints. We believe it would be helpful to first define the cellular degeneration caused by various mutations in deafness genes into the following four stages (Figure 1).

1. Stage I: deafness mutations in this category result in no detectable cellular degeneration in HCs, supporting cells and SGNs (Figure 2A) for a considerable period of time at the adult stage, but eventually degenerate. Treatment given for stage I SNHL may offer the best chance for intervention success via cochlear gene therapy. Possible genes and mutations in this category include claudin-11 (made from *CLDN11* gene) (Gow et al., 2004) and other auditory neuropathy spectrum of genes listed in Table 2 and Supplemental Table 2. It may be speculated that specific forms of age-dependent hearing loss (ADHL) may also belong to stage I, in which hair cells are relatively intact at the adult stage but under great apoptotic stress toward early degeneration.
2. Stage II: mutations in this category give rise to degeneration to only OHCs at the adult stage, but most inner hair cells (IHCs), supporting cells (SCs) and SGNs remain morphologically intact (Figure 2B, and also see Table 2). Gene therapy may significantly help SNHL at this disease stage by slowing down the degeneration process of hair cells or by rendering IHCs functional again. Our studies using *Gjb6*^−/−^ mice suggest mutations in this gene may have a slow degeneration time course and may below to this category (Sun et al., 2009).
3. Stage III: at this stage most inner and outer HCs are already degenerated. Supporting cells in the organ of Corti and significant portion of SGNs are relatively intact. Many common deafness genes (e.g., *Slc26a4, Tmc1*) that specifically affect HC or cochlear development (Tables 1, 2) may belong to this category.
4. Stage IV: at this stage only a layer of non-specific epithelial cells remain in the organ of Corti. All HCs, supporting cells and cells in the SV, and a significant portion of SGNs are degenerated. The degeneration in the middle and basal regions of the cochlea of conditional connexin26 knockout mice at the adult stage is a good example of stage IV degeneration in the cochlea (Wang et al., 2009). In our studies of a mouse model of Jervell Lange-Nielsen syndrome, we observed collapse of the Reissner's membrane and degeneration of multiple types of cochlear cells in the adult organ of Corti of *Kcnq1*^−/−^ mouse cochlea. We concluded that the optimal time window for the treatment of the *Kcnq1* null mutation would be before these permanent histological changes happen. Any therapy implemented after malformation of the cochlea would be significantly more difficult. Patients at advanced disease stages (e.g., stages III & IV) may theoretically benefit more from cell-based transplantation methods, if the mutation could be corrected *ex vivo* and transplanted back.

Most cochlear gene therapy studies use knockout mice as animal models, although some also used pharmacologically-damaged cochlea (Table 1 and Supplemental Table 1). Cochlear gene therapy has been evaluated in the prevention of hearing loss in mouse models of various forms, including genetic disorders of synaptic transmission of IHCs (Akil et al., 2012), failure in SV functions (Chang et al., 2015b), defect in auditory transduction of stereocilia (Pan et al., 2017), and dysfunctional supporting cells (Miwa et al., 2013; Yu et al., 2014). Some studies have shown that early postnatal intervention can result in hearing preservation to nearly WT level in these otherwise profoundly deaf animal models [e.g., vesicular glutamate transporter 3 null (*Vglut3*^−/−^), *Kcnq1*^−/−^]. Others demonstrate partial hearing preservation ranging from 10 to 30 dB. In addition to functional data, these studies generally also confirm significant alleviation of cellular degeneration in the cochlea when recombinant viral vectors were injected prior to the onset of degeneration in the organ of Corti in early postnatal stages. One important landmark that has as yet eluded cochlear gene therapies has been the capacity to restore hearing in the adult stage, when deafness has already occurred in animal models.

Results summarized in Table 1 raise questions about the nature of long-term effects of cochlear gene therapies, as many studies (Chang et al., 2015b; Kim et al., 2016; Isgrig et al., 2017) show only transient treatment effects in mouse models lasting from ~7 weeks to 6 months. Interestingly, conclusions about the long-term effects of ocular gene therapies are still unclear or otherwise controversial. In human follow-up studies from the LCA2 trials, it was reported that functional visual improvements persisted for up to 3 years after AAV2-RPE65 injection in LCA patients, although cellular degeneration in the retina continued to progress (Cideciyan et al., 2013; Testa et al., 2013; Bainbridge et al., 2015). After 3 years, vision improvement progressively diminished in patients (Bainbridge et al., 2015; Jacobson et al., 2015). It remains unclear why the effects of some human ocular gene therapy trials were limited to about 3 years. Some suggested that virally-expressed RPE65 may slowly decline to a level below the needed therapeutic threshold level 3 years after injection (Bainbridge et al., 2015). Continuing cellular degeneration, even in the presence of functional recovery, may be another key factor which precludes longer-term treatment efficacy. Treatments designed to virally express both the targeted WT gene and appropriate neurotrophic factor may prolong the efficacious period of treatment, although more tests are needed to examine whether this combination treatment is truly more effective.

Other important additions to the current state of knowledge include our understanding of the effects of virally-mediated ectopic expressions. Our studies using either *Kcnq1* (Chang et al., 2015b) or connexin knockout mice (Yu et al., 2014) indicated that ectopic expressions are very common when viral expressions are driven by a strong but generic promoter (e.g., CBA, CMV). At least in the short term (<3 months), ectopic expression appear to not adversely affect normal cochlear functions, as demonstrated by virally-mediated expressions of *Gjb2, Gjb6* (Yu et al., 2014), and *Kcnq1* (Chang et al., 2015b) in WT mice. Both ABR thresholds and cochlear morphology are indistinguishable between the treated and un-injected ears in WT mice. These results suggest that it is probably unnecessary to seek cell type-specific promoter in the viral construct (e.g., that drives gene expression which precisely matches the endogenous gene without any ectopic expression), although whether the ectopically expressed proteins have long-term harmful effects is unclear. More data are needed to answer whether silencing of generic viral promoters (e.g., CMV or CAG), or whether cell-type specific promoters is needed.

Another important piece of information we have learned relates to intracellular trafficking of virally expressed proteins. Our results show that virally-expressed exogenous protein is correctly trafficked to its native location (Yu et al., 2014; Chang et al., 2015b), as exogenous *Kcnq1* was correctly targeted exclusively to the apical membrane of marginal cells. Similar results are observed for virally-expressed *Gjb2*. The connexin protein expressed from viral particles is transported to the cell membrane and forms intercellular gap junctions both in *in vitro* (Sun et al., 2005) and *in vivo* studies (Yu et al., 2014). Studies using Usher mutant mice (Pan et al., 2017) again confirm that virally-expressed exogenous protein is correctly trafficked to the tip of stereocilia. These results suggest that crucial endogenous protein regulatory mechanisms govern the transport and assembly of virally-expressed proteins, and that the over-expressed protein can be trafficked as native proteins are and can be co-assembled with their native molecular partners to form functional membrane channels. These studies (Chang et al., 2015b; Gao et al., 2017; Pan et al., 2017) also revealed that, in the case of *Kcnq1* expression in the marginal cells, an expression in 61–75% of cells is sufficient to show significant treatment efficacy (Chang et al., 2015b). The percentage of cells expressing harmonin is found to be ~80% (Pan et al., 2017). In both studies hearing was significantly improved by about 50 dB. Whether higher transduction efficacy may give better or longer-lasting treatment effect is unknown.

Studies of ocular gene therapy have also suggested new directions for advancing cochlear gene therapy. Gene therapy of exudative macular degeneration has suggested an interesting design, which is not based on correcting specific gene mutations but rather on targeting the biological pathway to prevent neovascular pathology (e.g., Clinicaltrials.gov identifier NCT01494805 and NCT01678872, designed to virally express angiostatin and endostatin to prevent angiogenesis). The likely efficacy of this pathway-dependent approach for cochlear gene therapy has been suggested by over-expression studies using transgenic mice (Wang et al., 2011; Huang et al., 2013) in preventing both age- and noise-dependent hearing losses. As ADHL affects a large number of people (http://www.who.int/mediacentre/factsheets/fs300/en/), it would be very interesting to study whether gene therapies based on a design of biological pathways could be used here.

## Remaining major obstacles

A common focus of molecular/cell therapy studies for SNHL (Raphael et al., 1996; Derby et al., 1999; Shibata et al., 2009; Sacheli et al., 2012; Wang et al., 2013) is the regeneration of sensory hair cells with surviving supporting cells, or by using a cell replacement approach to preserve normal cochlear functions (Sacheli et al., 2012). We would argue that none of these approaches may be applied to treat significant portions of SNHL cases as they elide treatment of genetic root causes. Transforming surviving cells into hair cells is unlikely to help deaf patients suffering from genetic mutations because the causative mutation still remains in the genome. The new hair cells, even if successfully regenerated, will still suffer the consequences of the original genetic mutation that lead to severe degeneration (Figures 2C,D). In addition, cell therapies theoretically have higher risks of tumorigenesis associated with reprogramming of stem cells or immunological response to the transplanted stem cells. In addition, there is low likelihood that transplanted cells can survive in a degenerated organ of Corti (Wang et al., 2009). So far there is relatively little evidence that *ATOH1* transfection can produce substantial numbers of hair cells in adult animals (Brigande and Heller, 2009). It appears that cochlear gene therapy may be closer to clinical trials than cell therapies for treating SNHL.

As we make progress in biology-based therapies in animal models, researchers have also identified major obstacles which could hinder the successful translation of cochlear gene therapy into clinical applications. In addition to technical difficulties that need to be overcome, such as the size limitation to the transduction of many deafness genes larger than 5 kb, preclinical studies in animal models still need better answers about how to achieve stable and long-term treatment effects, and there is also a need to develop models of larger animals to demonstrate efficacy and a lack of adverse immune responses. A more challenging remaining issue, however, is that many deafness genes-including the most common ones (e.g., *GJB2, SLC26A4, TMC1*)-are developmentally-critical genes. These genes account for more than half of all cases of human genetic deafness. Null mutations of these genes generally lead to early degeneration of multiple types of cells in the cochlea (Wangemann et al., 2007; Holt et al., 2014; Chang et al., 2015a; Nishio et al., 2016). Studies of our lab (Chang et al., 2015a) as well as those of other investigators (Choi et al., 2011; Li et al., 2013) suggest that gene therapy interventions in humans for either connexin or pendrin null mutations may need embryonic gene delivery (virus injections) into the inner ear. There are severe degeneration and developmental interruptions in the cochlea of Cx26 null mutant mice before hearing starts. Degeneration stage in the adult cochlea is likely to reach either stage III or IV as defined in Figures 1C,D. Successful cases of ocular gene therapy indicate that morphological preservation of the cellular structure of the sensory organ is required for effective treatments (Dalkara et al., 2016). Severe morphological damage at the early stage renders therapeutic success of later stage intervention unlikely (Chang et al., 2015b).

If the goal for developing cochlear gene therapy is the treatment of common genetic deafness, one critical test for preclinical trials must be to demonstrate treatment efficacy for hearing restoration at the adult-stage after hearing loss has already occurred, which means we will have to deal with degenerated cochleae similar to those of CI patients that only rely on SGN survival. Can cochlear gene therapy be applied to those SNHL patients? Many insects use their auditory neurons as the first-order mechano-transduction (MT) apparatus for hearing (Kamikouchi et al., 2006; Coen and Murthy, 2016). Ectopic expressions of light-sensitive membrane proteins (termed as optogenetics method, Bi et al., 2006) in neurons have been used to control neuronal excitability including the auditory neurons (Hernandez et al., 2014). Application of optogenetics approach in ocular gene therapy has been advanced to clinical trials (e.g., NCT numbers: NCT02556736, NCT03326336) (Jacobson et al., 2013). Considering that there may be a significant limit to the number of cases of genetic deafness treatable by cochlear gene therapy, as we have analyzed above (Hoang Dinh et al., 2009; Askew et al., 2015; Chang et al., 2015a), here we propose a novel mechano-genetic approach (Figure 3). The idea is to transform SGNs to directly respond to the vibration of the basilar membrane by virally-expressing mechano-sensitive channels in these neurons. This new idea would need substantially lower requirements in terms of cell survival as compared to traditional gene therapies and would not require the use of any prosthetic devices. By design it requires only the survival of SGNs in the cochlea (Figure 3). No regeneration of IHCs or OHCs is needed, and it does not require the presence of endocochlear potential. These are the same thresholds for treatment as those required by CIs. The success of the approach, however, will rely on the induction of peripheral fibers of the SG neurons into the sensory epithelium of the cochlea (Figure 3) in order to better sense the vibration of the basilar membrane. Virally-mediated expression of exogeneous proteins in SGNs (Fukui and Raphael, 2013; Wang et al., 2013; Shibata et al., 2017) and the induction of its fibers into the sensory epithelium area are supported by published literature (Shibata et al., 2010, 2011). Identification of the best candidates and modification of mechanosensitive channels (Zhang et al., 2015) to have the appropriate sensitivity and response dynamics are needed for the success of mechano-genetic approach.

Novel technical advancements are still needed to accelerate the preclinical trials into human clinical trials. For example, it would be a great advantage to have a non-invasive biopsy method for the cochlea, similar to the optical coherence tomography (OCT) which has been proven to be indispensable in ocular gene therapy, for morphological evaluation at the cellular level for cochlear gene therapy. In addition, key steps and changes outside of the laboratory are critically needed. The success of cochlear gene therapy can benefit tremendously from efficient partnerships between academia research groups, pharmaceutical companies, federal and private funding agencies, and government policy makers. Collaborative translational efforts including the creation of open-access and multi-center databases of correlative phenotype-genotype information for diagnosis, standardized gene therapy protocols (preferably for adult-stage treatment of different cellular targets in the cochlea), establishment of meaningful outcome measures and major regulatory protocols, and better handling of the intellectual property issues in the best interests of all involved. The first report of successful ocular gene therapy in a large animal was reported (Acland et al., 2001) 16 years ago and first human trails were published about 10 years ago (Dalkara et al., 2016). FDA approved the first ocular gene therapy in December of 2017. Judging from this timeline, human applications of cochlear gene therapy may be still 20 years away. The presence of foundational supports as outlined above would very likely facilitate significant advancements and accelerate the translation of discoveries made in the laboratories into promising gene therapies for SNHL.

## Author contributions

All authors listed have made a substantial, direct, and intellectual contribution to the work, and approved it for publication. More specifically, WZ and XL designed and generated Figures 1–3, and made substantial contributions to the generation of all the Tables and Supplemental Tables. SK, WW, and CC made substantial contributions to the generation of all the Tables and Supplemental Tables.

### Conflict of interest statement

The authors declare that the research was conducted in the absence of any commercial or financial relationships that could be construed as a potential conflict of interest.
